# Patient involvement in qualitative data analysis in a trial of a patient‐centred intervention: Reconciling lay knowledge and scientific method

**DOI:** 10.1111/hex.12814

**Published:** 2018-08-02

**Authors:** Julia Frost, Andy Gibson, Faith Harris‐Golesworthy, Jim Harris, Nicky Britten

**Affiliations:** ^1^ University of Exeter Medical School Exeter UK; ^2^ Department of Health and Social Sciences University of West England Bristol UK; ^3^ PenCLAHRC Patient and Public Involvement Team University of Exeter Medical School Exeter UK; ^4^ University of Exeter Medical School University of Exeter Exeter UK

## Abstract

**Background:**

We conducted a pilot study of an intervention to facilitate patients’ agenda setting in clinical consultations. The primary aim of the study was to test the feasibility of running the randomized controlled trial. A secondary objective was to assess the extent to which patient and public involvement (PPI) could contribute to the process of qualitative data analysis (QDA).

**Aims:**

To describe a novel approach to including patient partners in QDA; to illustrate the kinds of contribution that patient partners made to QDA in this context; and to propose a characterization of a process by which patient involvement can contribute to knowledge production.

**Methods:**

Six patient and public representatives were supported to contribute to data analysis via a range of modalities. During a series of QDA workshops, experienced research staff role‐played consultations and interviews, and provided vignettes. Workshop data and PPI diaries were analysed using thematic discourse analysis.

**Results:**

We characterized a process of thesis, antithesis and synthesis. This PPI group contributed to the rigour and validity of the study findings by challenging their own and the researchers’ assumptions, and by testing the emerging hypotheses. By training PPI representatives to undertake qualitative data analysis, we transformed our understanding of doctor–patient consultations.

**Conclusions:**

This research required changes to our usual research practices but was in keeping with the objective of establishing meaningful patient involvement for a future definitive trial. This work was informed by concepts of critical humility, and a process of knowledge production enabled via the construction of a knowledge space.

## 1 INTRODUCTION

Enabling patient and public involvement (PPI) in health services research is now a prerequisite.^1^, ^2^, ^3^, ^4^ Two of us have theorized PPI as “knowledge spaces,” that is spaces where expert knowledge and forms of lay knowledge can interact with each other on an equal basis.^5^ In this paper, we trace out how the interaction between different forms of knowledge works in practice drawing on an example of PPI in qualitative data analysis to illustrate our points. Our analysis of the process has led us to conceptualize this interaction as a dialectical one, operating between lay and academic perspectives.

Previous work has indicated that PPI has a role in qualitative data analysis (QDA). It can correct researchers’ misinterpretations and challenge the ways in which findings are reported,^6^, ^7^ and add value to the products of research analysis.^8^ While qualitative methods to augment clinical trials are mainstream,^9^ the role of PPI is often overlooked.^10^, ^11^ Where PPI representatives have been involved in QDA alongside clinical trials, the impact of such involvement is often unclear.^12^

Examples of PPI in QDA as explicit emancipatory practice, sometimes described as community‐based participatory research (CBPR),^13^ are prevalent in sociological research and achieved by tailoring how data are presented, engaged with, managed and categorized. Working with disadvantaged women, Jackson^14^ provided facilitation alongside simplified instructions and processes, such as encouraging participants to summarize data in lay language. Conducting analysis with people with learning disabilities, Nind^15^ recommended tailoring approaches to local contexts, with academics variously taking on the role of “trainer, coach, scaffolder, mentor, partner in dialogue, co‐learner, reciprocal learner and practical facilitator.” Kaomea^16^ has also shown that indigenous participants work with a different set of perspectives and tools when undertaking analysis, which may challenge taken for granted research practices, allowing findings to be challenged and re‐examined.

The underpinning philosophies of CBPR and PPI are commensurate, with both valuing meaningful partnership and collaboration to achieve change in the conduct and products of research.^17^ We, academic co‐authors JF, AG, and NB, also drew on the literature about critical humility and participatory research to guide our conduct.^13^, ^18^ Emancipatory practices can be fostered by researchers’ “critical humility,” or lifelong commitment to self‐evaluation and critique, as lifelong learners and reflective practitioners.^18^ The political imperative is to subvert or challenge the status quo (here the canon of accepted research methods and practices), with the objective of enabling those who are traditionally the focus of studies (e.g. “patients”) to understand their world in order to transform it—a form of “catalytic validity”.^19^ Although Lather^20^ proposed that catalytic validity needs to be consciously designed into the research process in order to democratize knowledge, Baines^21^ contends that facilitating meaningful PPI contributions to knowledge would require a shift in the political economy of universities and research funders. Furthermore, the selectivity with which PPI representatives are chosen (reifying some voices to the detriment of others) has also been questioned.^22^, ^23^ However, this situation is beginning to change.^24^, ^25^

In this study, as the aim was to ensure that patients’ concerns were addressed in the consultation, a correspondingly emancipatory approach to the data analysis required patients’ perspectives to inform the data analysis. Crucially, analytic judgements about whether the intervention had indeed facilitated the discussions of patients’ concerns should be informed by patient perspectives. Conventionally, these judgements have been made by academic researchers alone.^26^

### 1.1 Diabetes Intervention for Agenda Trial (DIAT)

We conducted a pilot study of a pre‐consultation intervention in which patients were supported by health‐care assistants (HCAs) to complete a Web‐based intervention to facilitate the production of “their own agenda” for discussion in a consultation with their diabetologists (Diabetes Intervention for Agenda Trial (DIAT);^27^, ^28^). By combining trial and qualitative methods, we sought to identify how the intervention influenced consultations, and decide whether proceeding to a full trial was appropriate.

Patients were involved at all stages, in order to increase the probability that the intervention would be adopted in practice.^29^ The research question was generated from a prioritization exercise, undertaken by the National Institute for Health Research (NIHR) Collaboration for Leadership in Applied Health Research and Care in the South West Peninsula (PenCLAHRC) with active involvement from the Peninsula Patient and Public Involvement Group (PenPIG). People with diabetes (including PPI representative 1 or “PPI1”) suggested that outpatient clinic appointments are pressured times, where health professionals can overlook issues that are worrying patients or where patients can feel inhibited from voicing their concerns. Supported by a PPI Research Fellow (AG), two members of PenPIG (FH‐G and JH) who have diabetes joined the research team as funding co‐applicants and members of the project management team. They co‐wrote the study documentation, assisted with training the HCAs to deliver the pre‐consultation intervention, participated in the data analysis and disseminated preliminary findings. Eight additional people with diabetes, recruited from the NIHR Diabetes Research Network and Diabetes UK, provided feedback on an early iteration of the intervention, and another participated in the trial steering group. With 4 other members of PenPIG, the two co‐applicants (“PPI2” and “PPI3”) participated in a PPI qualitative data analysis group (“PPI group” with R denoting Researcher).

Seventy‐one patients were randomized to either the intervention or usual care.^26^ With participants’ and diabetologists’ consent, we audio‐recorded intervention sessions and clinical consultations, to explore how participants generated and utilized the intervention. Thirteen patients randomized to receive the intervention consented to be recorded and twelve had both an intervention session and a consultation recorded, while one had only their consultation recorded. Twelve control patients consented and had a “usual” consultation recorded. Recording consultations facilitated our understanding of the impact of the production of an agenda on the clinical consultation, and its subsequent use in practice when compared with usual care.^24^, ^30^ Author A conducted 12 semi‐structured interviews with a convenience sample of participating staff and 12 patients (6 in each trial arm) to explore wider organizational factors. Interview questions were based upon the existing literature and PPI perspectives and aimed to capture staff and patient experience of diabetes consultations and the intervention specifically. Data were transcribed verbatim, anonymized and managed using Nvivo software. Preliminary framework analysis^31^ facilitated deductive and inductive analysis, enabling the exploration of the role of the intervention and trial processes alongside the elicitation of patient, provider and PPI perspectives.

Qualitative data analysis began with JF and a diabetes specialist nurse (DSN) co‐applicant independently familiarizing themselves with the first 20 consultations.^32^ In the control arm, we explored the *usual* consultation style of each participating diabetologist. In the intervention arm, we explored the extent to which diabetologist used the agenda form as a guide for the consultation. A preliminary charting exercise (Table 1) allowed us to identify relationships between the process of agenda identification and agenda use.^23^ Thus JF and the DSN co‐applicant identified a preliminary typology of consultations (patient ignored, diabetologist led, patient led, diabetologist and patient led).

**Table 1:** Extract from preliminary charting exercise

Demographic data	Intervention	Agenda form	Consultation
Patient: XXXX Diabetologist: XX Allocation: *Intervention* Facilitator: XX Age: XX Gender: X Date: xx.xx.xx	F provided information and support as per training. P struggles to read the screen. Needed text enlarged and moved chair closer. P volunteers that he has problems with spelling ‘I suffer from…I can't spell, so’, and F spells out ‘blood sugar’… [long pause] P: “Last time, no, the time before, I saw [Consultant]. At the end of the consultation he said to me, erm, ‘you have 10 seconds is there anything else you would like to ask me’ which I thought was, you know, ha ha ha…couldn't believe it, I was so gob smacked… I just walked out you know…Well he seemed very arrogant to me… Can I make a comment on it here?” F reiterates that P can write what he wants, but he does not make this comment. Ps concerns would ‘take pages’ because he has ‘any number of them’ — he says that he is ‘not trying to be facetious’ and ‘asks ‘how long have I got?’ as his diabetes has such an impact: ‘Physically can't do what I used to do… I can't eat what I used to do I can't drink…I can't…It completely alters your way of life, you know.’	*Particular concern:* “my blood sugar levels are always high i find that emotions play a big part in raising these levels can I increase the dosage of exenatide” *Concern about diabetes:* “there are so many ways it alters your there are too many to mention” *Concern about medication: “*can i increase exenatide what good will it do how much should i increase the insulin dose by to lower the sugar levels” *Concern about managing diabetes:* “diet”	No initial discussion of agenda form. D begins by identifying P on his records and checking which medication P is taking. P is slightly confused, and D says: ‘I suppose one thing this illustrates is really useful if you can bring a copy of your prescription with you.’ P says that he recently had a heart attack, but D cannot find a record of relevant blood tests, and D asks P if he is ‘quite sure’ of when he was in hospital. D says that a recent HbA1c result ‘wasn't very good’ and P admits that he has been struggling with self‐monitoring, and has been having high results, which he attributes to stress about his sisters illness. D then refers to the agenda form: “Okay now you have got this list of questions here for the study and the first thing you ask is my blood sugar levels are always high I find the emotions play a big part in raising these levels. Can I increase the dose of Exenatide? The answer to that last question is no, you can't.” There is a lengthy discussion re: Exenatide, with D proposing other medications to control HbA1c, and P suggesting that D has not got his dose correct

The aims of this study were to describe a novel approach to involving patient partners in QDA; to illustrate the kinds of contribution that patient partners made to QDA; and to propose a characterization of a process by which patient involvement can contribute to knowledge production.

## 2 METHODS

### 2.1 Training

JF, AG, and NB trained a group of six PPI representatives (the PPI group, including co‐applicants PPI2 and PPI3), in QDA techniques (such as paying attention to nuances in consultations, and constructing patterns within and across consultations^32^) during three workshops. During the workshops, the PPI group had access to verbatim transcripts of consultations and in‐depth interviews, but data governance considerations meant that data access was confined to the workshop. The PPI group did not listen to the audio‐recordings of the transcripts directly, to maintain confidentiality. JF and AG therefore role‐played the anonymized consultations from the charting exercise, making sure that they captured the audible nuances of the audiotape, and provided vignettes which summarized the “cases.” Role‐play provides insight into social interaction and can provide a stimulus for discussion.^33^ Vignettes are stories about individuals, situations and structures, which allow actions to be explored in context and provide a less personal means of exploring sensitive topics.^34^

**Figure 1:**
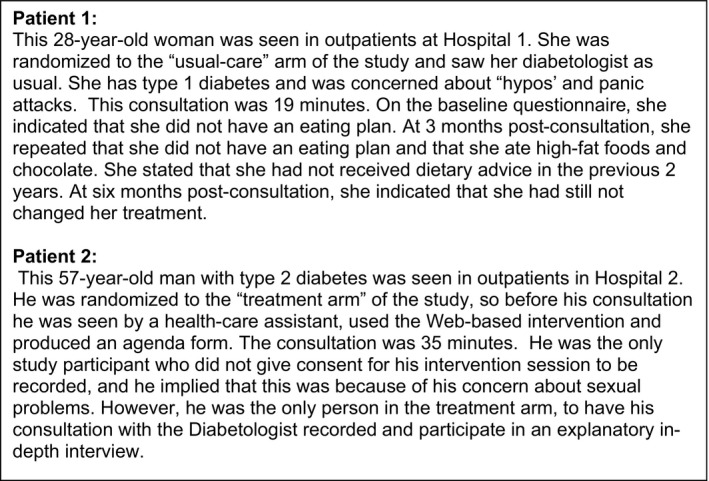
Illustrative vignettes

In *Workshop 1* (3 hours), the researchers introduced the study to the PPI group members who were unfamiliar with the project and the aims of the workshops. They described data collection and preliminary analysis to date. Large data matrices were used to demonstrate how the researchers had conducted their preliminary coding, and participants were given instructions as to how they might produce their own codes about “what is going on” and “whose agenda is dominant” from the data that they would be given. The researchers then provided the PPI group with a vignette (Patient 1—Figure 1) and asked them to read the transcript and observe a role‐play of a consultation in the usual care arm of the trial. PPI participants were then provided with the researchers’ preliminary typology, as a stimulus to develop their own codes. The researchers had produced a form to guide participants in conducting their analysis, but this was felt by the group to be too constraining. Instead, “codes” were identified from line‐by‐line discussion of the transcript. These analytical discussions were captured by the researchers using a flip chart. The skilled PPI facilitator summarized PPI discussions and probed further for clarification. Notes of these discussions informed revisions to the preliminary coding frame, which, in turn, were fed back to the PPI group and shaped their subsequent analysis.

In *Workshop 2* (6 hours), role‐play was used to portray a consultation in the intervention arm of the trial (Patient 2—Figure 1) and a subsequent qualitative interview in which the participant discussed his experience, alongside a contextual vignette. The PPI group read and discussed transcripts from both the consultation and interview, and were asked to critique and develop the typology. In *Workshop 3* (2 hours), the PPI group was provided with their revised typology, with the codes that they had identified, supported with descriptors from the previous discussions. They were given 4 further vignettes from a purposive sample of the consultation data. In small groups, participants led the analytical discussions, testing the codes and descriptors against the data, in the same way that teams of qualitative researchers conduct analytical discussions.

Thus, over the course of three workshops, and using a hands‐on approach, the researchers and the PPI group went through the key stages of framework analysis, consisting of familiarization with the data, coding, developing an analytical framework, applying the framework, charting and interpreting the data.^31^, ^33^

### 2.2 Data collection

The 2 PPI co‐applicants (PPI2 and PPI3) kept diaries and included information which they felt comfortable sharing with the wider research team.^35^ Repeat semi‐structured interviews (2 qualitative interviews, one in each year of the project, with each of the two co‐applicants), conducted by the PPI facilitator (AG), elaborated upon the reflections captured in the diaries and changes in experience over time.^36^ PPI co‐applicants were asked about their experience of participation, their identification of barriers and facilitators to participation, and their perceptions of the value that their contribution made to the research. The QDA workshops were audio‐recorded. Ethical approval for the original proposal and subsequent amendments was granted by the NHS Research Ethics Committee North West—Preston (13/NW/0123). The 2 PPI co‐applicants provided written consent for their diaries and interviews to be analysed. As the group's PPI analysis was a PPI activity to shape research, NHS REC approval was not required.

### 2.3 Data analysis

Thematic discourse analysis was developed over the course of 3 workshops using all sources of data, paying attention to micro‐level discourse and the construction of the meaning of involvement to illustrate the cumulative process of knowledge production in a knowledge space^31^, ^32^ by Authors JF, AG, and NB, with further clarification from FH‐G and JH. The results of each workshop are presented in turn, reflecting our analysis at both a workshop and cumulative level, and our objective of assessing the contribution of PPI to the process of QDA.

## 3 RESULTS

### 3.1 Workshop 1: Hypothesis testing

By providing the PPI group with a preliminary typology, the academic co‐authors were demonstrating an analytical process without suggesting that the PPI group should validate those suggestions.^37^ In terms of the critical humility of the researchers, and recognizing that our preliminary analyses were led by our own assumptions, we saw this as part of a conversation in which we were opening ourselves up to their experiential knowledge and interpretations as one would with other research and clinical partners.^38^ The PPI group subsequently developed the preliminary typology by proposing the terms “*patient identified*” (rather than “*patient led*”) and “*diabetologist facilitated”* (rather than *“diabetologist led”*) as more nuanced, with subsequent rich discussions forming coding descriptors. With regard to “*patient ignored*,” the PPI group were able to describe the challenge of voicing the patients’ agenda in usual care by referencing what was different in the intervention consultations:

PPI2 In the [example from usual care], I would have been on my own; I was well *left*, you know, I was not part of it at all. This [example from intervention arm] I think he was joining in, he was involved, he was led a bit, but he was saying ‘Yes’, so he was obviously led in the right direction. (Extract 1)

Similarly, a *“diabetologist facilitated”* style was identified in both trial arms; such that a diabetologist's particular practice could both pre‐date and be enhanced by the use of an agenda form:

PPI4 In the control arm, having a consultant who typically has a facilitative style [without agenda form] e.g. [Patient from control arm] talks about erectile dysfunction comfortably with the same consultant that [Patient from intervention arm] does. (Extract 2)

By comparing the interview data with the consultation data, the PPI group were able to discern aspects that the “*patients identified”* (e.g. wrote down on the agenda form without the need to vocalize sensitive matters), and which patients later discussed in an interview:

PPI5 Yeah, I think the consultant did quite a good job really, and I think he also handled talking about erectile dysfunction [item on agenda form which patient did not articulate] very sympathetically and very, you know¸ sort of a way that perhaps hopefully was comfortable with the patient, too, which is, you know, sometimes, I think things like that are handled very poorly. (Extract 3)

Finally, the PPI group discussed what constituted a “*patient identified and diabetologist facilitated”* agenda:

PPI6 I think, yeah, the doctor brought his agenda into play but he interweaved it into the patient's agenda. He fed in at the important places with his stuff. (Extract 4)

These newly identified codes and descriptors were then used by the research team to further analyse the data and develop display matrices for each category:

Diabetologist facilitated Rather than seeing the consultations as ‘*Diabetologist led’* (implicitly paternalistic), the PPI group recognised and described a consultative style that was facilitative. This could occur in the usual care arm of the study, where an agenda form was not produced, but when a diabetologists skilfully elicited the patient's concerns.

Patient identified (intervention arm) Rather than assuming that the agenda form in the intervention arm would empower the patient (implicitly beneficial), the PPI group explained that it was sufficient and appropriate for the patient to produce an agenda to convey their concerns, with the diabetologists subsequently leading the consultation.

These nuanced codes are in keeping with the findings of Cribb et al^39^ who suggest that broader conceptions of shared decision‐making allow for open‐ended and fully dialogic ways of relating. These new codes were then applied to the whole data set and complete matrices discussed again with the PPI group.

### 3.2 Workshop 2: Challenging

Here, we explore one of the coding discussions (“*patient identified and diabetologist facilitated”*), to demonstrate that the PPI group were able to develop a refined typology of the consultations which challenged the assumptions of the project team, as well as their own views. The group were discussing how to code a particular consultation where a diabetologist used a patient's agenda form to frame the consultation:

PPI4 I got it when [consultant] said about the two agendas merging, I got that bit. If I was the patient I would think I would be happy‐ish to hear that, you know, at least we're going the right way.

PPI3 Who *cares* about [consultant's] agenda, I'm here with *my* agenda! I've just worked on, brought it in, I don't really care what his agenda is [laughs] I want my issues dealt with, you know. I mean, if somebody said that to me: well, whoopee do for you! Have I got what I want? (Extract 5)

PPI3 was emphatic that “her agenda” should always be prioritized over that of the diabetologist. The discussion continued, as to when it would be appropriate for a diabetologist to question the patient's agenda:

PPI3 I did wonder why the [consultant] didn't probe more into why the patient didn't want four injections a day…

R You could also interpret that positively…if the patient has stated a preference they would like it to be that way, it's not up to me to question that, it's up to me to discuss whether we can actually…

PPI3 I don't think I agree with that at all…there's such an issue here about, you know, blood sugar control is so important for all the other things including cardiovascular disease. (Extract 6)

Despite wanting her agenda to be *enacted*, PPI3 thought it remiss of the diabetologist not to challenge the patient's underlying assumptions:

PPI3 I think if you're going to be a good physician, then you have to recognise when you need to step in regardless of the patient's agenda and I think it's very good that he acknowledges the patient's agenda and he's following the patient's agenda but on this point I think that he needs to explore it more with a view to giving this man the best possible treatment he can have… But definitely I think, you know, that may be on his agenda, but sometimes I think that they — the consultant should be overriding it with as much negotiation as is possible.

R So you said if I go I don't care about the consultant's agenda, it's my agenda.

PPI3 Yes.

R What you've just been saying, actually, the consultant should override the patient's agenda if…

PPI3 Only in that situation…I don't think, I can't think of another scenario. (Extract 7)

In this way, the group collectively challenged each other about assumed rules for consultation participation and caveats for the use of an agenda, and were able to move from a typology of patients and professionals, to scenarios when a patient's agenda may or may not be appropriate.^39^ This particular thread developed into a discussion of whether a diabetologist should prioritize health concerns over a patient's desire for quality of life, and PPI2 shared his experience of multimorbidity and polypharmacy:

PPI2 I balance it — I really want a better quality of life. At the moment I'm not getting it and my main thing is… it's like the morning medication: fifteen tablets that I take and I can't eat anything for at least an hour and a half because I feel sick, you know, and that's not a good quality of life.

R So would you want more‐ a kind of explicit discussion about the quality of life that maybe if you took fewer medicines or if it was changed whether that might bring increased risks, you know, because in a way, that would be up to you. What kind of a balance would you want between quality of life and risks of things getting worse?

PPI2 I think, yeah, I would like that sort of discussion, yeah… (Extract 8)

The group members questioned PPI2 about his medication and he conceded that he valued his consultant being directive:

PPI2 My consultant… said to me…if we don't do something you won't be here in twelve months, you know, so it was drastic for me…. The drugs I'm on, and I've been on them so long, it's, you know, the damage is already done, you know. I may probably have a limited amount of life left but I know what I can do and how long, do you know what I mean? I know my limitations. I don't think this guy knows his limitations. (Extract 9)

After PPI2 was so open about his own experience, PPI3 revised her own position again:

PPI2 Actually I'm just going to confound all of my arguments…With PPI3 talking I've just remembered something about quality of life over health care, and at one point my consultant and I agreed that if I had any more insulin I would put on more weight, I would develop more insulin resistance and blood sugars would go up and go up. So we actually agreed, despite the fact that my HbA1c was quite high, that having more insulin at that point was just going to make all of my problems worse. But then, looking at it back down the line, that's probably why I've got retinopathy, you know. I've lost a lot of my sight on the periphery, can't drive at night… (Extract 10)

This account demonstrates the importance of trust and continuity between patients and providers and the importance of continuity of care; but also the importance of trust and continuity within the PPI group, such that difficult conversations could be surfaced, tensions explored, and misunderstandings or assumptions resolved. The contrasting and complementing views of the PPI group acted as a brake on any assumption that the researchers might have made about the patient's agenda always having primacy, and the quality of their argument augmented knowledge about the contexts in which an agenda form may or may not be useful.

### 3.3 Workshop 3: Re‐challenge

In the final workshop, members of the PPI group demonstrated their ability to analyse and synthesize the findings from the qualitative data. This illustration depicts the confidence with which they could apply their understanding (or analytical framework) to vignettes produced from additional consultations and interviews:

PPI6 Well we've got a 42 year old diabetic Type 1 who would appear to have been visiting a diabetic consultant and wasn't very excited about being there, answered all the questions negatively, didn't commit anything that he wanted to improve and out of nowhere the consultant said about changing the insulin regime but didn't quantify it, and then he said ‘Oh, I'll quite happily do that because of me brother.’ So there's a note in there which he never picked up on ‘Why, what's wrong with your brother?’ and he'd just died, he was a diabetic and he'd died of some cardiac problem. Er, and at the end of it, you know, it was just another meeting he's been to out of his 84 over 42 years that didn't go anywhere. And, you know, he's going through the motions for them… Yes, it's *consultant led*, you can identify that from the script here to point to consultant led, yes, there are still, um, the *agenda unvoiced*, yes, there's a limit in there. (Extract 11)

Members of the PPI group were given a further opportunity to amend the analytical typology, and it was concluded that the data fitted the categories earlier identified by the PPI representatives. Finally, the group were able to produce a highly nuanced synthetic account of best practice (and new knowledge):

R What's good practice here?

PPI4 I think it should depend on the patient and, to me, it should be ‐ appear in different [analytical] boxes with different patients, ‘cause everybody's not the same. And everybody won't be able to initiating it, you know, some might just be going in and waiting for the doctor to actually start it off and ask them the questions like we did with one of them.

PPI2 And based purely on that individual, an individual patient.

PPI3 I suppose in an ideal world, with the same consultant, different patients; you would hope to see the columns populated differently. (Extract 13)

In this final example, the PPI group identified that diabetes consultations required care that was tailored to the needs of the patient and contextual, suggesting that an agenda form was helpful in some situations but not others—a potential challenge to the premise of the feasibility study. Thus, the PPI contribution and wider analysis of the DIAT feasibility study suggested that the study protocol would need further development before a definitive randomized controlled trial would be warranted. By equipping the PPI group with analytical techniques, they were able to engage with the data and crucially, in this clinical trial of a patient‐centred intervention, make the findings clinically relevant.

## 4 DISCUSSION

There have been calls both to standardize the practice of involving participants in clinical trials^40^ and the reporting of involvement in qualitative analyses.^41^ We have responded by demonstrating the process which was undertaken by the PPI group, facilitated by experienced qualitative researchers (JF and NB) and skilled PPI facilitation (AG). By applying our in‐depth knowledge of qualitative research theory and PPI practices, we were able to improve the inclusivity and understanding of this PPI group,^42^ by developing an approach more akin to the emancipatory and participatory practices described in the sociological literature.^14^, ^15^, ^16^

To facilitate catalytic validity, defined as the degree to which research moves those it studies to enable them to better understand the world and transform it,^19^ the academic researchers reflected on their own practices and attempted to foster mutually beneficial and non‐paternalistic partnerships with members of the PPI group.^18^ This required the deconstruction of established qualitative practices, to enable the PPI representatives to participate in the interpretation of data and knowledge production. This experience led us to reflect that the emancipatory or ethical imperative of PPI is not at odds with the scientific methods employed in clinical trials (of testing and refining hypotheses).

In trying to understand and conceptualize the process described above, both for ourselves and others, we came to the conclusion that what had taken place could be best understood as a dialectical interaction made possible by the creation of a knowledge space where people with differing social relationships to the research process,.that is patients and academics could, nevertheless, interact. In health care, professionals are in possession of a particular form of cultural capital (accumulated knowledge, skills and behaviours) based on their qualifications, and similar privilege applies for health services researchers in the arena of research.^5^ Previous research has identified how non‐research–based professionals can feel isolated by the process of having specific tasks within a trial, which can inhibit their sense of agency.^43^ Clinicians have suggested that lack of time allowed by planned research activities has limited their ability to adequately recruit patients to clinical trial targets,^44^, ^45^ while others have detailed the emotional labour of managing patients’ (mis)understanding of randomization processes.^46^ Even though the PPI activities in this study were crafted to achieve longitudinal inclusion, this did not mitigate the partial view of the research that PPI collaborators expressed early in the research process. We thus considered the experience as akin to the boundaries of inclusion described by some clinicians participating in trials. However, by making changes to our usual research practices, the contribution to QDA specifically was perceived by PPI collaborators was a particularly valuable experience, because it went some way towards valuing their knowledge and redistributing power, which enabled them as “knowledgeable actors” within a knowledge space.^5^ In turn, this enabled us to develop a far more nuanced understanding of diabetology consultations and identify the limitations of our intervention within current health‐care provision.^27^, ^28^

In 1808, Fichte^47^ identified that forming a thesis involves setting out or laying down an argument. The thesis is then challenged by its antithesis. This challenge is then itself challenged, and a process of reconciliation is sought in a synthesis where both views are accommodated or surpassed. In an ongoing process, this synthesis becomes the starting point for a new cycle of knowledge creation.^48^ For Fichte, this process was not the preserve of academics, but rather antithesis was the process by which others held the elite in check.^49^ In 1940, Popper^50^ contended that this dialectic triad (of thesis, antithesis and synthesis) surpassed the traditional scientific method of trial and error, as it contained an imperative to reconcile or further explore different perspectives, which were absent from other methods of scientific enquiry.

A *thesis* is formed when an idea or *hypothesis* is outlined, and we began our analytical process by orientating the PPI group to our preliminary typology in response to the overall research question which the pilot trial sought to answer. The PPI group subsequently developed the preliminary typology by proposing their own terms. An *antithesis* is the *challenge* to a thesis by the presentation of contrasting or opposing views, and as the PPI group discussed the data with each other, they began to challenge the researchers’, each other's, and indeed their own interpretations. With such a wealth of experience, we did not expect the PPI group to have a unified voice, and here, we demonstrate how they wove individual stories into storylines which, as qualitative researchers, we typically think of as “themes.” A *synthesis* occurs when the *challenge produced by an antithesis is itself challenged*. It becomes the next thesis to inform the analytical process, which the PPI group engaged with by testing out their own hypotheses. In the final analysis workshop, members of the group demonstrated their ability to analyse and synthesize the findings from the qualitative data. This approach may have utility in other areas of health services research, and clinical trials more specifically, for example when assumptions about the acceptability or effectiveness of trial interventions warrants further exploration to identify core components and their optimal alignment.^29^

By detailing our approach, we are able to suggest a template for how other researchers might facilitate PPI in QDA (Table 2). We are aware that this is one illustrative example of a broader dialectical process. Here, the PPI group was provided with a typology to initiate the dialectical process, although this initial “thesis” could have been generated by patients, with academics providing the “antithesis.” Similarly, the dialectic process could have continued, but we were constrained by the timeframe of the funded project.

**Table 2:** Template for PPI involvement in QDA

Objectives	Activities
Overall objective: For PPI representatives to make a contribution to QDA	Establish nature of involvement. Recruitment from a range of organizations. Study orientation: Familiarization with aims and objectives. Introduction to wider team members. Ongoing jargon busting. Attendance at team/steering meetings
To learn from the experiences of PPI collaborators and make any necessary modifications to practice	Completion of PPI dairies and initial interviews, to identify barriers and facilitators to participation, and experience more generally
To improve researcher understanding	Facilitate PPI input into: (as appropriate) Study documentation, including recruitment materials, letters to potential participants, lay summaries. Intervention development Staff training. Qualitative topic guide.
To provide stimulus for analytical discussions	Provisional coding frame produced by research team members
For PPI to make a contribution to QDA For QDA to be accessible To capture PPI perspectives	Workshop 1: Training Role‐play of consultations/interviews Provision of vignettes Data collection techniques: audio‐recording, note‐taking, flipchart, emails
Revision of coding frame as data collection and data analysis develop	Revision of coding frame by researcher, (revision of topic guide/research practices could also be revised here)
For PPI to make a contribution to QDA For QDA to be accessible To capture PPI perspectives	Workshop 2: Role‐play of consultations/interviews Provision of vignettes Data collection techniques: audio‐recording, note‐taking, flipchart, emails
Revision of coding frame as data collection and data analysis develop	Revision of coding frame by researchers
Finalization of coding frame once all data have been accounted for	Workshop 3: Provision of vignettes
To learn from the experiences of PPI collaborators and plan future revisions to practice	Evaluation: Repeat interviews and revisit diaries Data collection techniques: audio‐recording, note‐taking, flipchart, emails
To include PPI representatives in the dissemination of the research findings	PPI co‐authorship on draft papers: PPI input into paper writing and wider dissemination activities

A strength of this approach is that it facilitates meaningful patient and public involvement which we feel both augments the validity of the research findings and provides a replicable process that could be employed in other clinical trials or qualitative studies. A limitation might concern the individuals who were engaged in this study. Two members of the research team had diabetes, but we supplemented this involvement with workshops involving a broader group of people. This enabled us to surface tensions between perspectives of different PPI representatives, enabling us to develop a more nuanced understanding of the issues raise which contributed to a shared understanding of the research findings. A further limitation might be the skill and training required by the research team to facilitate PPI in this way,^51^, ^52^ which may be perceived as burdensome. This requires an understanding of the principles, practices and possibilities afforded by the various qualitative methodologies, as well as openness to the challenges of translating the theory and practice of research in order to make it accessible and participatory,^53^, ^54^ rather than as a quick‐fix or tickbox approach.

## 5 CONCLUSION

We successfully trained and collaborated with PPI representatives to undertake qualitative data analysis which transformed our understanding of doctor–patient consultations,^24^ and the appropriate uses of an agenda form. This required changes to our usual research practices, in terms of purposively constructing a knowledge space to break down lay and professional boundaries; but was in keeping with the objective of establishing meaningful patient involvement for a definitive trial. We propose that our knowledge production process of thesis, antithesis and synthesis can be used as a template for other researchers who work on clinical trials or health services research more generally. Furthermore, we suggest that researchers should document and analyse the nature of the interpretation itself.

We contend that the driver for actively involving patients and members of the public in clinical trials should not merely be its requirement for successful funding.^38^ Rather, the imperative should be to develop more holistic understandings of complex problems via a change in the way that knowledge is produced,^48^ Dingwall et al^47^ proposed that qualitative research can augment the findings of clinical trials and address the humanitarian issues of equity and effectiveness. We propose that by undertaking QDA with PPI representatives using a dialectic process, we collectively produced more valid and clinically relevant findings.

## References

[1] National Health and Medical Research Council and Consumer's Health Forum of Australia . Statement on Consumer and Community Participation in Health and Medical Research 2016 https://www.nhmrc.gov.au/guidelines-publications/s01. Accessed February 7, 2018

[2] Canadian Institutes of Health Research . Citizen Engagement Framework 2010 http://www.cihr-irsc.gc.ca/e/41753.html. Accessed February 7, 2018

[3] Patient Protection and Affordable Care Act . Public Law. 2010 https://www.gpo.gov/fdsys/pkg/PLAW-111publ148/pdf/PLAW-111publ148.pdf. Accessed February 7, 2018

[4] National Institute for Health Research . Patient and Public Involvement. 2016 http://www.invo.org.uk/resource-centre/resource-for-researchers/. Accessed February 7, 2018

[5] Gibson A , Britten N , Lynch J . Theoretical directions for an emancipatory concept of patient and public involvement. Health. 2012;16(5):531‐547. 22535648 10.1177/1363459312438563

[6] Staley K . Exploring Impact: Public Involvement in NHS, Public Health and Social Care Research. Eastleigh: Involve; 2009.

[7] Brett J , Staniszewska S , Mockford C , Seers K , Herron‐Marx S , Bayliss H . The PIRICOM Study: A Systematic Review of the Conceptualisation, Measurement, Impact and Outcomes of Patients and Public Involvement in Health and Social Care Research. London: UKCRC; 2010.

[8] Garfield S , Jheeta S , Husson F , Jacklin A , Norton C , Franklin BD . Lay involvement in the analysis of qualitative data in health services research: a descriptive study. Res Involv Engagem. 2016;2:29. 29507764 10.1186/s40900-016-0041-z PMC5831865

[9] Donovan J , Mills N , Smith M , et al. Improving design and conduct of randomised trials by embedding them in qualitative research: ProtecT (prostate testing for cancer and treatment) study. BMJ. 2002;325(7367):766‐770. 12364308 10.1136/bmj.325.7367.766 PMC1124277

[10] O'Caithan A , Thomas K , Drabble S , Rudolph A , Hewison J . What can qualitative research do for randomised controlled trials? A systematic mapping review BMJ Open. 2013;3:e002889. 10.1136/bmjopen-2013-002889 PMC3669723 23794542

[11] Drabble S , O'Caithan A , Thomas K , Rudolph A , Hewison J . Describing qualitative research undertaken with randomised controlled trials in grant proposals: a documentary analysis. BMC Med Res Methodol. 2014;14(24):1‐11. 24533771 10.1186/1471-2288-14-24 PMC3937073

[12] Gamble C , Dudley L , Allam A , et al. An evidence base to optimise methods for involving patient and public contributors in clinical trials: a mixed‐methods study. Health Services Deliv Res. 2015;3:39. 26378330

[13] Minkler M , Wallerstein N , eds. Community Based Participatory Research for Health. San Francisco, CA: Jossey‐Bass Publishers 2003.

[14] Jackson S . A participatory group process to analyze qualitative data. Prog Community Health Partnersh. 2007;2(2):161‐170. 10.1353/cpr.0.0010 20208250

[15] Nind M . Participatory data analysis: a step too far? Qualitat Res. 2011;11(4):349‐363.

[16] Kaomea J . Qualitative analysis as Ho'oku'iku'i or bricolage: teaching emancipatory indigenous research in postcolonial Hawai'i. Qual Inq. 2016;22(2):99‐106.

[17] Boote J , Wong R , Booth A . ‘Talking the talk or walking the walk?’ A bibliometric review of the literature on public involvement in health research published between 1995 and 2009. Health Expect. 2007;18:44‐57. 10.1111/hex.12007 PMC5060762 23033933

[18] Tervalon M , Murray‐Garcia J . Cultural humility versus cultural competence: A critical distinction in defining physician training outcomes in multicultural education. J Health Care Poor Underserved. 1998;9(2):117‐125. 10073197 10.1353/hpu.2010.0233

[19] Kincheloe JL , McLaren P . Rethinking critical theory and qualitative research In: Trueba ET, Zou Y, eds. Ethnography and Schools: Qualitative Approaches to the Study of Education. Lanham, MD: Rowman and Littlefield; 2002:87‐138.

[20] Lather P . Issues in validity in openly ideological research: between a rock and a soft place. Interchange. 1986;17(4):63‐84.

[21] Baines D . The case for catalytic validity: building health and safety through knowledge transfer. Policy Pract Health Safety. 2007;5(1):75‐89.

[22] Martin G . ‘Ordinary people only’: knowledge, representativeness, and the publics of public participation in healthcare. Sociol Health Illn. 2008;30(1):35‐54. 18254832 10.1111/j.1467-9566.2007.01027.x

[23] Fredriksson M , Tritter J . Disentangling patient and public involvement in healthcare decisions: why the difference matters. Sociol Health Illn. 2017;39(1):95‐111. 27862007 10.1111/1467-9566.12483

[24] Bagley H , Short H , Haman N , et al. A patient and public involvement (PPI toolkit for meaningful and flexible involvement in clinical trials – a work in progress. Res Involve Engagem. 2016;2:15. 10.1186/s40900-016-0029-8 PMC5611579 29062516

[25] INVOLVE . Guidance on co‐producing a research project. 2018 http://www.invo.org.uk/wp-content/uploads/2018/03/Copro_Guidance_Mar18.pdf. Accessed 21.03.18

[26] Barry C , Bradley CP , Britten N , Stevenson FA , Barber N . Patients’ unvoiced agendas in general practice consultations: qualitative study. BMJ. 2000;320:1246‐125022. 10797036 10.1136/bmj.320.7244.1246 PMC27368

[27] Frost J , Anderson R , Argyle C , et al. A pilot randomised controlled trial of a preconsultation web‐based intervention to improve the care quality and clinical outcomes of diabetes outpatients (DIAT). BMJ Open. 2013;3:e003396. 10.1136/bmjopen-2013-003396 PMC3731775 23903815

[28] Ukoumunne OC , Vaidya B , Frost J , et al. A preconsultation web‐based tool to generate an agenda for discussion in diabetes outpatient clinics to improve patient outcomes (DIAT): a feasibility study. BMJ Open. 2017;7:e013519. 10.1136/bmjopen-2016-013519 PMC5353257 28270389

[29] Ennis L , Wykes T . Impact of patient involvement in mental health research: Longitudinal study. Br J Psychiatry. 2013;203(5):381‐386. 24029538 10.1192/bjp.bp.112.119818

[30] Collins S , Britten N , Ruusuvuori J , Thompson A . Patient Participation in Health Care Consultations. Berkshire: Open University Press; 2007.

[31] Ritchie J , Lewis J , McNaughton Nicholls C , Ormston R . Qualitative Research Practice: A Guide for Social Science Students and Researchers. London: Sage; 2013.

[32] Miles M , Huberman A , Saldana J . Qualitative Data Analysis: A Sourcebook, 3rd edn London: Sage; 2014.

[33] Adams J , Mabusela M . Assessing with role play: an innovation in assessment practice. J Soc Sci. 2014;41(3):363‐374.

[34] Barter C , Renold E . The Use of Vignettes in Qualitative Research In: Social Research Update, 25, University of Surrey, 1999 http://sru.soc.surrey.ac.uk/SRU25.html

[35] Corti L . Using diaries in social research In: Social Research Update. 2. University of Surrey, 1993 http://sru.soc.surrey.ac.uk/SRU2.html

[36] Britten N . Qualitative Interviews In: Pope C, Mays N, eds. Qualitative Research in Health Care. 3rd ed Oxford: Blackwell Publishing 2006:12‐20

[37] Ocloo J , Matthews R . From tokenism to empowerment: progressing patient and public involvement in healthcare improvement. BMJ Quality Safety. 2016; 25:626‐632. 10.1136/bmjqs-2015-004839 PMC4975844 26993640

[38] Britten N , Denford S , Harris‐Golesworthy F , Jibson S , Pyart N , Stein K . Patient involvement in drug licensing: A case study. Soc Sci Med. 2015;131:289‐296. 25454636 10.1016/j.socscimed.2014.10.024

[39] Cribb A , Entwistle V . Shared decision making: trade‐offs between narrower and broader conceptions. Health Expect. 2011;14:210‐219. 21592264 10.1111/j.1369-7625.2011.00694.x PMC5060567

[40] Evans B , Bedson E , Bell P , et al. Involving service users in trials: developing a standard operating procedure. Trials. 2013;14(219):1‐7. 23866730 10.1186/1745-6215-14-219 PMC3725161

[41] Staniszewska S , Brett J , Mockford C , Barber R . The GRIPP checklist: Strengthening the quality of patient and public involvement reporting in research. Int J Technol Assess Health Care. 2011;27(4):391‐399. 22004782 10.1017/S0266462311000481

[42] Koniotou M , Evans B , Chatters R , et al. Involving older people in a multi‐centre randomised trial of a complex intervention in pre‐hospital emergency care: implementation of a collaborative model. Trials. 2015;16(298):1‐10. 26156174 10.1186/s13063-015-0821-z PMC4496939

[43] Ziebland S , Featherstone K , Snowdon C , Barker K , Frost H , Fairbank J . Does it matter if clinicians recruiting for a trail don't understand what the trial is really about? Qualitative study of surgeons’ experiences of participation in a pragmatic multi‐centre RCT? Trials. 2007;8:4. 17257440 10.1186/1745-6215-8-4 PMC1794540

[44] Tognoni G , Alli C , Avanzini F , et al. Randomised clinical trials in general practice: lessons from a failure. BMJ. 1991;303:969‐971. 1954424 10.1136/bmj.303.6808.969 PMC1671349

[45] Prout H , Butler C , Kinnersley P , Robling M , Hood K , Tudor‐Jones R . A qualitative evaluation of implementing a randomized controlled trial in general practice. Fam Pract. 2003;20(6):675‐681. 14701891 10.1093/fampra/cmg609

[46] Lawton J , Kirkham J , White D , Rankin D , Cooper C , Heller C . Uncovering the emotional aspects of working on a clinical trial,: a qualitative study of the experiences and views of staff involved in a Type 1 diabetes trial. Trials. 2015;16:3. 25566971 10.1186/1745-6215-16-3 PMC4326295

[47] Fichte J . Wissenschaftslehre, Nova methodo. Edited and translated by Breazeale, D., (1998) Foundations of Transcendental Philosophy. Ithaca, NY: Cornell University Press 1808.

[48] Wilden A . Montage analytic and dialectic. Am J Semiot. 1984;3(1):25‐47.

[49] Qvortrup M . Johann Gottlieb Fichte (1762‐1814). Philosophy Now. 2014;104:50‐52.

[50] Popper K . What is dialectic? Mind. 1940;49(196):403‐426.

[51] Van Staa A , Jedeloo S , Latour J , Trappenburg M . Exciting but exhausting: experiences with participatory research with chronically ill adolescents. Health Expect. 2009;13:95‐107. 19682098 10.1111/j.1369-7625.2009.00574.x PMC5060512

[52] Dudley L , Gamble C , Allam A , et al. A little more conversation please? Qualitative study of researchers’ and patients’ interview accounts of training for patient and public involvement in clinical trials. Trials. 2015;16:190. 25928689 10.1186/s13063-015-0667-4 PMC4410574

[53] Dingwall R , Murphy E , Watson P , Greatbatch D , Parker S . Catching goldfish. J Health Serv Res Policy. 1998;3(3):167‐172. 10185376 10.1177/135581969800300308

[54] Rose D . Patient and public involvement in health research: Ethical imperative and/or radical challenge? J Health Psychol. 2014;19(1):149‐158. 24058120 10.1177/1359105313500249

